# Epidemiology of moderate-to-severe respiratory syncytial virus infections in children in subtropical Okinawa, Japan: a 4-year retrospective study

**DOI:** 10.1186/s41182-025-00824-3

**Published:** 2025-11-11

**Authors:** Kahoru Fukuoka–Araki, Kotaro Araki, Hiromi Fukuoka, Yoshiaki Cho, Kei Matayoshi, Tomoko Makiya, Saori Kinjo, Tetsu Yamashiro

**Affiliations:** 1 Department of Bacteriology, Graduate School of Medicine, University of the Ryukyus, 1076 Kiyuna, Ginowan City, Okinawa 901-2720 Japan; 2 Department of Pediatrics, Okinawa Chubu Hospital, 281 Miyazato, Uruma City, Okinawa 904-2293 Japan; 3 Department of Pediatrics, Yaeyama Hospital, 584-1 Maezato, Ishigaki City, Okinawa 907-0002 Japan; 4 Department of General Pediatrics, Okinawa Prefectural Nanbu Medical Center & Children’s Medical Center, 118-1 Arakawa, Shimajiri District, Haebaru Town, Okinawa 901-1193 Japan; 5 Department of Pediatrics, Okinawa Miyako Hospital, 427-1 Hirara Shimozato, Miyakojima City, Okinawa 906-0013 Japan; 6 Division of Pediatric Infectious Diseases, Department of Pediatrics, Okinawa Prefectural Nanbu Medical Center & Children’s Medical Center, 118-1 Arakawa, Shimajiri District, Haebaru Town, Okinawa 901-1193 Japan; 7 Department of Neonatology, Okinawa Chubu Hospital, 281 Miyazato, Uruma City, Okinawa 904-2293 Japan

**Keywords:** Respiratory syncytial virus, COVID-19, Respiratory infection, Pediatric infectious disease, Okinawa islands, Subtropical region

## Abstract

**Background:**

Respiratory syncytial virus (RSV) is a leading cause of acute lower respiratory tract infections (ALRTIs) in infants and young children worldwide. While its epidemiology is well-characterized in temperate climates, data from subtropical regions such as Okinawa, Japan, remain limited. This study aimed to describe the clinical and demographic characteristics, risk factors, and seasonality of moderate-to-severe RSV infections in children under 5 years across Okinawa.

**Methods:**

This retrospective, multicenter study analyzed pediatric cases of laboratoryconfirmed RSV infection requiring hospitalization between April 2017 and March 2021. Data were collected from four core hospitals across Okinawa Prefecture. Patients were categorized as having moderate or severe disease based on ICU admission status. Demographic variables, underlying diseases, household and childcare characteristics, and seasonal trends were assessed.

**Results:**

A total of 1541 hospitalized RSV cases were included, of which 117 (7.6%) were classified as severe. Overall, 89.0% were under 24 months of age, with the highest burden in the 0–2 month group. In univariate analysis, severe cases were significantly younger, more likely to have siblings, and less likely to attend nursery school compared with moderate cases. The overall prevalence of underlying diseases did not differ between groups; however, having two or more underlying diseases was significantly associated with severity. Multivariate logistic regression confirmed younger age, the presence of siblings, and underlying diseases (both any and multiple) as independent risk factors for severe infection. Seasonal peaks occurred consistently in summer during 2017–2019. In contrast, in 2020, coinciding with the onset of the COVID-19 pandemic, the epidemic curve became broader and peak timings varied across hospitals. Overall, the total number of cases decreased by 62% compared with the pre-pandemic average.

**Conclusions:**

This study provides a comprehensive region-wide assessment of moderate-to-severe pediatric RSV infections in a subtropical setting in Japan. Despite not including data on the use of palivizumab, nirsevimab, or maternal vaccination, the findings provide essential baseline data to guide the implementation of new preventive strategies tailored to local epidemiology.

## Background

Respiratory syncytial virus (RSV), a member of the genus *Orthopneumovirus* in the family *Pneumoviridae*, is an enveloped, nonsegmented, negative-sense RNA virus and a leading cause of acute lower respiratory tract infections (ALRTIs) in infants and young children worldwide [1, 2]. Globally, RSV is responsible for approximately 33 million ALRTIs episodes annually, leading to more than 3 million hospitalization and an estimated 118,200 deaths among children under 5 years of age, with the majority occurring in low- and middle-income countries [2]. High-risk groups (HRGs) for moderate-to-severe RSV infection include preterm infants, children with chronic lung disease or congenital heart disease, immunocompromised individuals, and those with genetic conditions such as Down syndrome [3]. Severe RSV infection in early infancy has also been associated with long-term respiratory sequelae, including recurrent wheezing and increased risk of asthma in childhood [4, 5].

The prevalence and seasonality of RSV infection are strongly influenced by climatic factors. In temperate regions, RSV epidemics typically peak during the winter months, from September to January in the northern hemisphere, coinciding with cold and dry conditions [6, 7]. In contrast, tropical and subtropical regions, characterized by high humidity and rainfall, often experience year-round RSV activity with less distinct seasonal peaks [6].

In Japan, RSV is recognized as a leading cause of pediatric hospitalization for acute respiratory infections. Nationwide surveillance estimates the annual incidence of RSV infection at about 1,761 per 100,000 children under five years old [8]. Additionally, surveillance data from 2008 to 2015 reported 16–36 deaths each year due to RSV infection, although the total number of deaths showed a decreasing trend over time [9]. The country’s pronounced climatic gradient between northern and southern regions results in substantial variation in RSV epidemic timing nationwide [10].

Preventive strategies against RSV infection have advanced in recent years. In Japan, palivizumab has been available since 2002, primarily for preterm infants and those with congenital heart or chronic lung disease; however, its use is limited to HRGs  and requires monthly injections during the RSV season [11, 12]. Recently, nirsevimab, a long-acting monoclonal antibody providing season-long protection with single dose, was approved in Japan in 2024, although its insurance coverage is currently restricted to HRGs [13, 14]. Maternal RSV vaccination has recently become available in Japan; however, it is not covered by insurance and is generally administered on a self-pay basis rather than as part of the routine immunization program. Together, these developments highlight the need for robust baseline epidemiological data to guide the implementation of regionally tailored prevention strategies.

Continuous monitoring of regional RSV activity and issuing timely, region-specific public health alerts are considered critical for effective disease control. In Japan, RSV surveillance is carried out through sentinel pediatric sites across the country, which report weekly case and demographic data to health authorities. The Japan Institute for Health Security (JIHS) collects and analyzes these data [15], although clinical severity and outcomes are not included.

Okinawa Prefecture is separated from mainland Japan and consists of the main Okinawa Island as well as the Miyako and Yaeyama islands. It has a subtropical to tropical climate. Due to its geographical isolation, most residents receive medical care within the prefecture, and inter-prefectural patient transfer is uncommon, making Okinawa an ideal setting for comprehensive regional epidemiological studies. This study is unique in that it analyzes moderate-to-severe RSV infections across all of Okinawa prefecture, a subtropical region of Japan where seasonal patterns differ from the mainland and are more similar to those in Southeast Asia. By focusing on clinically significant cases, this work provides insights into risk factors and prevention strategies particularly relevant for this region.

## Methods

### Study setting and population

**Fig. 1:**
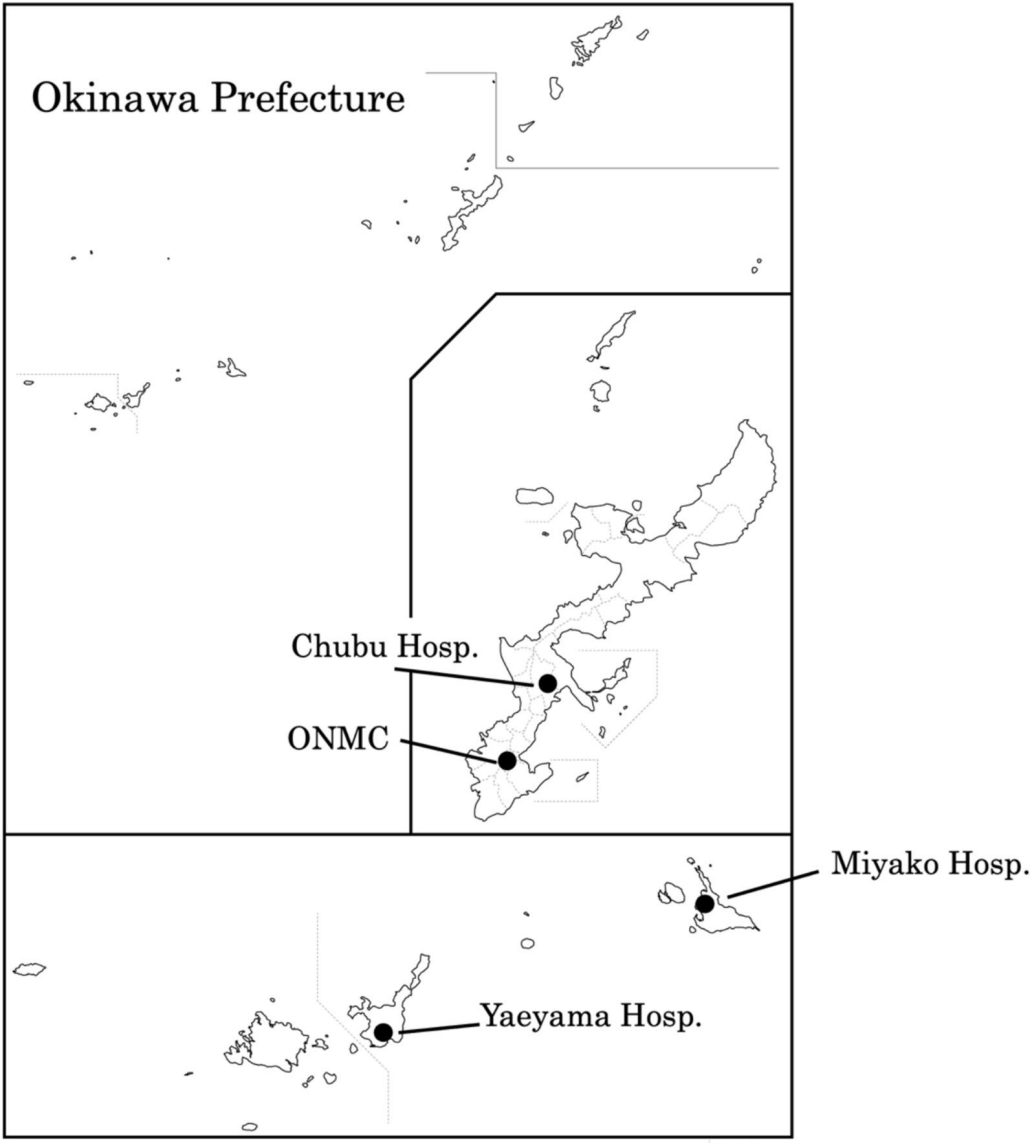
Map of Okinawa Prefecture showing the Okinawa main island along with the Miyako and Yaeyama Islands. The Okinawa main island lies approximately 1600 km southwest of Tokyo, while the Miyako and Ishigaki Islands are located about 300 km and 400 km from the main island, respectively. The locations of the four participating hospitals (ONMC, Chubu, Miyako, and Yaeyama) are indicated

Okinawa Prefecture, located approximately 1600 km southwest of Tokyo, comprises the Okinawa main island and multiple remote islands, including the Miyako and Yaeyama island groups situated about 300 km and 400 km further southwest, respectively (Fig. 1). The region has a subtropical to tropical oceanic climate, with an average maximum temperature of 29.1 °C in July and an average minimum of 17.3 °C in January, and total annual precipitation of 2,161 mm over the 5 year period beginning in 2020 [10]. The prefecture has a total population of approximately 1.5 million people, including roughly 76,800 children under 5 years of age. Population distribution is concentrated on the main island of Okinawa and its surrounding islands, which account for 92.4% of the residents, while the Miyako and Yaeyama Islands comprise 3.81% and 3.75%, respectively [16]. For healthcare administration, Okinawa Prefecture is divided into five medical service areas (MSAs) based on clusters of municipalities. On the main island, three MSAs—Northern, Central, and Southern—cover 9–16 adjacent municipalities each. The Miyako and Yaeyama MSAs correspond to the Miyako and Yaeyama Islands, respectively.

### Study design and participating hospitals

This is a retrospective, medical chart-based epidemiological study conducted over a four-year period from April 2017 to March 2021 at four regional core hospitals in Okinawa Prefecture: Okinawa Nanbu Medical Center & Children’s Medical Center (ONMC), Chubu Hospital, Miyako Hospital, and Yaeyama Hospital. ONMC (444 beds), located near Naha City, serves the Southern MSA, which accounts for approximately 54.5% of the main island population. Chubu Hospital (559 beds), located in Uruma City, serves the Central MSA, covering approximately 38.0% of the main island population. Miyako Hospital (276 beds) and Yaeyama Hospital (302 beds) are the sole core hospitals serving the Miyako and Yaeyama MSAs, respectively (Fig. 1).

### Patient enrollment and case classification

This study included children under 5 years of age who were hospitalized with RSV infection. RSV infection was diagnosed using nasopharyngeal swab specimens collected from children admitted with acute respiratory symptoms. Samples were tested by immunochromatographic assay with commercially available rapid antigen detection kits routinely employed in pediatric practice in Japan. The BD Veritor System for Rapid Detection of Respiratory Syncytial Virus (RSV) (256046) was used at ONMC, the ImunoAce RSV Neo Rapid Test Kit for Respiratory Syncytial Virus (RSV) (IARS0930) at Chubu and Miyako Hospitals, and the ALSONIC RSV Rapid Immunochromatographic Test Kit for Respiratory Syncytial Virus (22700AMX00679000) at Yaeyama Hospital. Although no unified research protocol was applied, all hospitals followed national standard clinical practices, ensuring consistency in diagnostic approaches across sites. Patients admitted exclusively to a general pediatric ward were classified as moderate cases, whereas those requiring intensive care unit (ICU) management were classified as severe cases. Indications for ICU admission were based on standard clinical criteria used at each hospital, including the need for high-flow oxygen therapy or mechanical ventilation, recurrent apnea, hemodynamic instability requiring intensive monitoring or intervention, or significant neurological complications. While exact admission thresholds may vary slightly among hospitals, all participating centers adhere to these widely accepted criteria, and ICU admission was determined by the attending physicians.

### Data collection

Clinical and demographic data were extracted from medical records, including age, sex, presence of siblings, nursery school attendance, preterm birth (gestational age < 37 weeks), underlying diseases, ICU admission, type of respiratory support, and discharge outcomes. Respiratory support modalities were recorded cumulatively, as individual patients could receive multiple interventions during hospitalization.

### Ethics approval

This retrospective study analyzed anonymized medical records. Consistent with Japan’s Ethical Guidelines for Life Sciences and Medical Research Involving Human Subjects, an opt-out process was used by posting a study announcement on the participating hospitals' websites, giving patients and families the opportunity to decline participation. The study was approved by the Clinical Research Review Committee of Okinawa Chubu Hospital, Japan (Approval No. 2020-68).

### Statistical analysis

Continuous variables were summarized as medians with interquartile ranges (IQRs) and compared using the Mann–Whitney U test. Categorical variables were presented as counts and percentages and analyzed using the chi-square test or Fisher’s exact test, as appropriate. Multivariate logistic regression analysis was performed for major factors, and odds ratios (ORs) with 95% confidence intervals (CIs) were calculated. All statistical analyses were performed using EZR, a graphical interface for R (Saitama Medical Center, Jichi Medical University). A two-sided *p* value of < 0.05 was considered statistically significant.

## Results

### Patients

A total of 1,561 records of children under 5 years of age with moderate-to-severe　RSV infection were initially enrolled. After excluding 20 cases due to failure to meet the case definition or duplication, 1,541 cases were included in the final analyses. Of the 1,541 cases, 548 were reported from ONMC, 522 from Chubu Hospital, 169 from Miyako Hospital, and 302 from Yaeyama Hospital. The 1,541 children hospitalized with RSV infection were classified into two groups: those with severe cases requiring ICU care and those with moderate cases not requiring ICU admission. A comparative analysis was performed between the two groups with respect to demographic characteristics, presence of siblings, nursery school attendance, preterm status and corresponding gestational age, and the presence and type of underlying diseases (Table 1).

**Table 1:** Background of Patients with Moderate-to-Severe RSV Infection

	Moderate	Severe	Total
Case	1424	117	1541
Median age, month (IQR)	10 (3–16)	3^*^ (1–11)	9 (3–16)
Female (%)	640 (44.9)	63 (53.8)	703 (45.6)
Presence of siblings (%)	936/1225 (76.4)	98/111 (88.3)^*^	1034/1336 (77.4)
Attending nursery school (%)	613/1120 (54.7)	42/105 (40.0)^*^	655/1,225 (53.5)
Preterm birth (%)	138 (9.7)	10 (8.5)	148 (9.6)
≤ 28 weeks	44	2	46
29–35 weeks	73	3	76
36 weeks	21	5	26
Underlying diseases overall (%)	206 (14.5)	22 (18.8)	228 (14.8)
Single	169	15	184
Respiratory (R)	80	7	87
Cardiovascular (CV)	23		27
Neurological (N)	20	4	21
Allergic (A)	15	0	15
Chromosomal (Ch)	11	0	11
Renal (Rn)	5	0	5
Hematologic and Oncologic (HO)	2	0	2
Endocrine and Metabolic (EM)	2	1	3
Others (Oth)	11	2	13
Two or more	37	7^*^	44
CV + Ch	7	1	8
R + N	6	1	7
R + Ch	3	2	5
R + CV	4	1	5
CV + N	4	0	4
R + A	3	0	3
R + EM	2	0	2
R + Oth	2	0	2
A + Rn	1	0	1
Rn + HO	1	0	1
R + CV + Ch	1	0	1
CV + Ch + EM	1	0	1
CV + Ch + Oth	1	0	1
R + N + A	1	0	1
R + CV + Oth	0	1	1
R + CV + N + Ch + Oth	0	1	1

^*^Significantly different from moderate RSV infection (*p* < 0.05)

IQR: Interquartile Range

In the univariate analysis, severe cases were significantly younger, more likely to have siblings, and less likely to attend nursery school compared with moderate cases. Among 1541 hospitalized children, 148 (9.6%) were preterm: 46 at ≤ 28 weeks, 76 at 29–35 weeks, and 26 at 36 weeks. Severe cases occurred in 2 (4.3%), 3 (5.2%), and 5 (11.4%) of these groups, respectively; however, no statistically significant difference was observed between the moderate and severe groups. The overall prevalence of underlying diseases did not differ significantly between the two groups (14.5% vs. 18.8%, *p* = 0.22); however, having two or more underlying diseases was more common among severe cases (*p* = 0.044). The distributions of individual underlying diseases and their combinations were also examined, but no significant differences were found between the two groups (Table 1).

In the multivariate logistic regression analysis, younger age and the presence of siblings remained significant independent risk factors for severe RSV infection. In addition, the presence of underlying diseases was significantly associated with severity: both the total presence of underlying diseases (OR 2.12, 95% CI 1.13–3.96, *p* = 0.019) and having two or more diseases (OR 5.34, 95% CI 1.90–14.95, *p* = 0.001) were identified as strong risk factors. Nursery school attendance and preterm birth were not significant predictors after adjustment, and female sex showed only a borderline association (Table 2).

**Table 2:** Multivariate logistic regression of risk factors for severe RSV infection

Variable	OR	95% CI	*p*-value
Median Age, month	0.94	0.90–0.97	< 0.001
Female	1.51	0.99–2.30	0.054
Presence of siblings	2.17	1.16–4.09	0.016
Attending nursery school	1.13	0.65–1.95	0.670
Preterm birth	1.12	0.53–2.37	0.769
Underlying disease: Overall	2.12	1.13–3.96	0.019
Underlying disease: ≥ 2	5.34	1.90–14.95	0.001

### ***Seasonal and regional distribution of moderate***-***to***-***severe RSV infections***

**Fig. 2:**
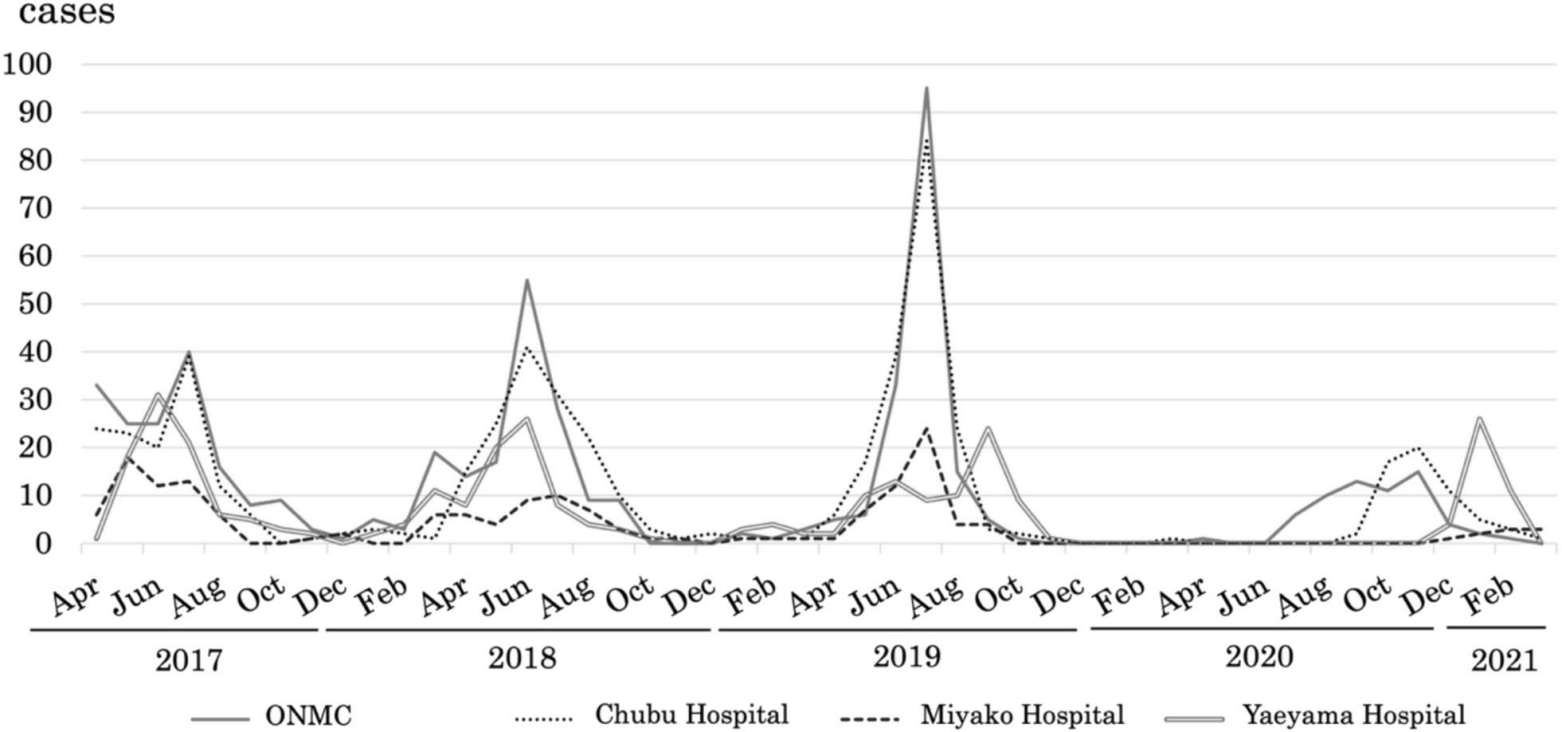
Seasonal distribution of moderate-to-severe RSV cases across four hospitals in Okinawa Prefecture, April 2017–March 2021. From 2017 to 2019, clear summer peaks (June–August) were observed, with minimal activity in December–February; peaks in Miyako were less distinct and broader than in other regions. In 2020, coinciding with the COVID-19 pandemic, the epidemic curve broadened and peak timings varied among hospitals (ONMC: Aug–Nov; Chubu: Nov–Dec; Yaeyama: Jan–Feb), while Miyako showed no clear peak

Figure 2 shows the seasonal distribution of moderate-to-severe RSV cases across four hospitals in Okinawa Prefecture from April 2017 to March 2021. From 2017 to 2019, clear seasonal peaks were consistently observed during the summer months (June–August), while RSV activity remained minimal between December and February. This pattern was evident in most regions; however, in the Miyako region, the peaks were less distinct, broader, and lower compared to other areas. The peak incidence also gradually increased over this period. In contrast, in 2020, coinciding with the onset of the COVID-19 pandemic, the epidemic curve became broader and peak timings varied among hospitals: ONMC observed its peak in August–November, Chubu in November–December, and Yaeyama in January–February, while Miyako did not show a clear seasonal peak　(Fig. 2).

### Age distribution of moderate-to-severe RSV infections in children under 24 months

**Fig. 3:**
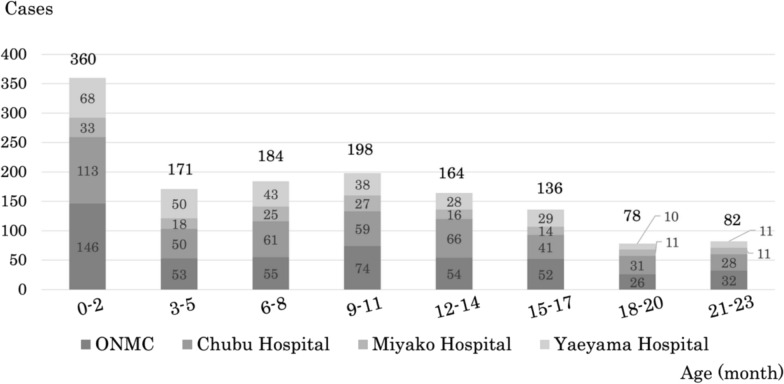
Age distribution of moderate-to-severe RSV infections in children under 24 months. Among 1,373 cases, the 0–2 month group accounted for the largest proportion (26%), followed by a secondary peak in the 9–11 month group. Cases declined progressively after 12 months of age, with no notable regional differences observed

Among the 1,541 patients with moderate-to-severe RSV infection, 89.0% (*n* = 1,373) were under 24 months of age. The age distribution in three-month intervals is shown in Fig. 3. The largest subgroup was the 0–2 month age group, accounting for 26% (*n* = 360) of cases. The number of cases dropped sharply in the 3–5 month group, increased again in the 6–11 month groups with a secondary peak at 9–11 months, and then declined steadily through 21–23 months. These patterns were consistent across all regions, with no notable regional differences observed (Fig. 3).

### ***Comparison of age***-***specific proportions of moderate***-***to***-***severe RSV cases before and during COVID***-***19***

**Fig. 4:**
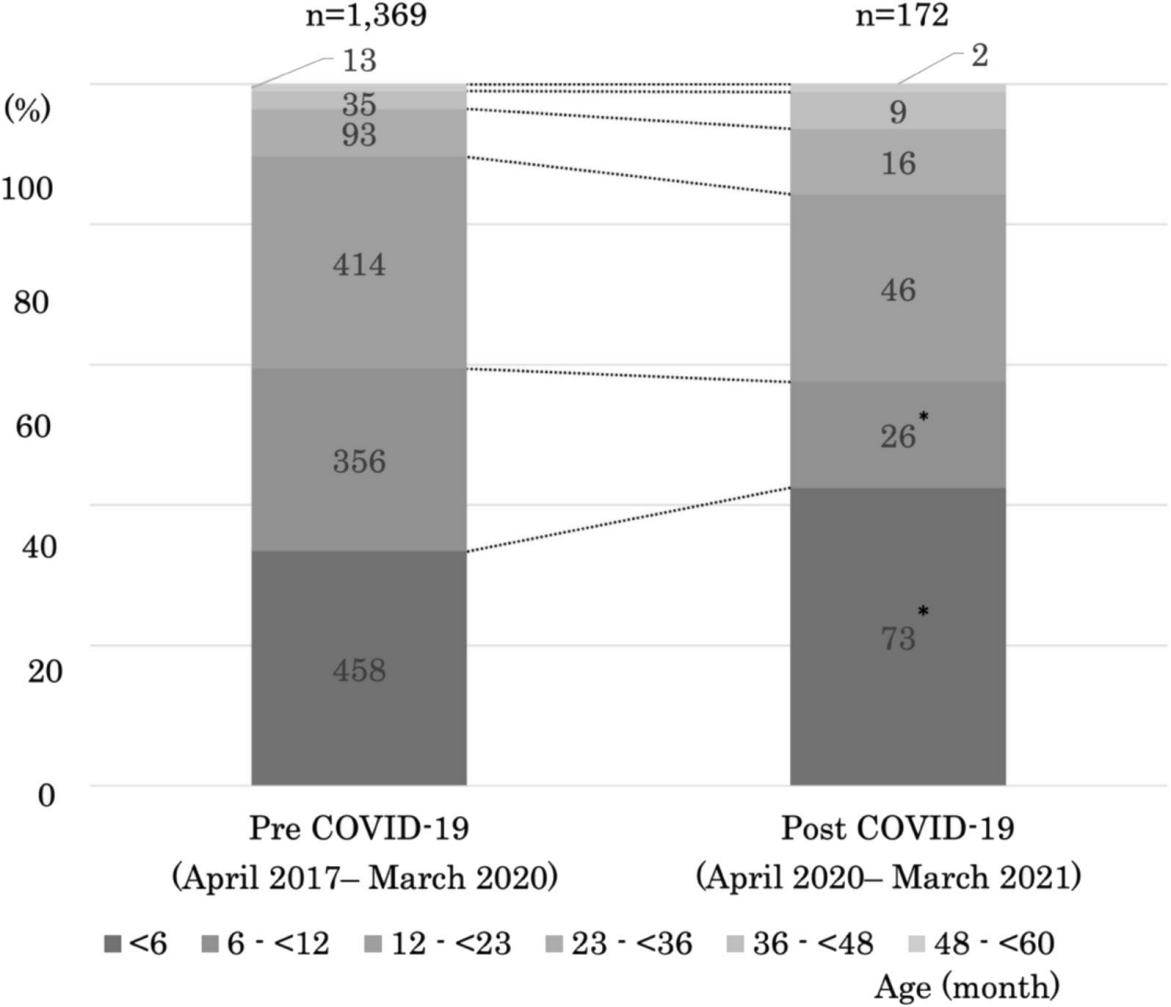
Age-stratified distribution of children under 5 with moderate-to-severe RSV infection before (2017–2020) and during (2020–2021) the COVID-19 pandemic. A total of 1,369 cases occurred pre-pandemic (approximately 456 annually) and 172 during the pandemic, representing a 62% decrease. In both periods, infants < 6 months accounted for the largest proportion, which increased significantly during the pandemic, while cases in the 6– < 12 month group declined. No significant changes were seen in children ≥ 12 months. *statistically significant difference (*p* < 0.05)

The age-stratified distribution of children under 5 with moderate-to-severe RSV infection was analyzed for the periods before and during the pandemic. From April 2017 to March 2020, 1,369 cases were reported (approximately 456 annually), whereas only 172 cases occurred during April 2020–March 2021, representing a statistically significant 62% decrease from the pre-pandemic average. In both periods, infants aged < 6 months accounted for the largest proportion of cases; this proportion increased significantly in the pandemic period, while the proportion of infants aged 6 to < 12 months decreased significantly. There were no significant differences between periods for children aged ≥ 12 months (Fig. 4).

### ***Respiratory support and clinical outcomes in patients with moderate***-***to***-***severe RSV Infection***

Of the 1,541 patients with moderate-to-severe RSV infection, 1,039 (67.4%) required some form of respiratory support. Normal flow oxygen support via facial mask or nasal cannula was the most frequently used modality, administered to 998 patients (64.8%), including 898 moderate cases (63.1%) and 100 severe cases (85.5%). High-flow nasal cannula was used in 314 patients (20.3%), comprising 204 moderate cases (14.3%) and 110 severe cases (94.0%). Mechanical ventilation was provided to 52 patients (3.4%), including 3 moderate cases (0.2%) and 49 severe cases (41.9%). Respiratory support was not required in 501 patients (32.5%), all of whom were moderate cases. Overall, 1,538 patients (99.8%) recovered without complications. Complications occurred in three patients, all in the severe group. Two of these patients needed long-term mechanical ventilation, and one developed a rare condition called RSV-associated acute encephalopathy with biphasic seizures and late reduced diffusion (AESD). This patient was diagnosed with AESD based on the pattern of seizures and specific MRI findings, including widespread reduced diffusion in the brain's white matter, which showed a “bright tree appearance.” This patient subsequently experienced developmental delays. None of the three patients with complications had underlying diseases. No deaths were reported (Table 3).

**Table 3:** Respiratory support and Outcomes in patients with Moderate-to-Severe RSV infections

	Moderate, *n* = 1424 (%)	Severe, *n* = 117 (%)	Total, *n* = 1541 (%)
Respiratory support (cumulative)			
Normal flow O_2_ support (facial mask)	898 (63.1)	100 (85.5)^*^	998 (64.8)
High-flow Nasal Cannula O_2_ support	204 (14.3)	110 (94.0)^*^	314 (20.3)
Tracheal Intubation & Mechanical Ventilation	3 (0.2)	49 (41.9)^*^	52 (3.4%)
No O_2_ support	501 (35.2)	0 (0.0)^*^	501 (32.5)
Outcomes			
Recovered without Complications	1,424 (100)	114 (97.4)	1,538 (99.8)
Recovered with Complications	0	3 (2.6)^#^	3 (0.2)
Died	0	0	0 (0)

^*^Significantly different from moderate RSV infection (*p* < 0.05)

^#^Two required long-term mechanical ventilation, and one developed AESD with developmental delays

## Discussion

This is a comprehensive study examining the demographic and clinical characteristics of children hospitalized with moderate-to-severe RSV infection in Okinawa over a four-year period starting in April 2017. During the study period, the global COVID-19 pandemic led to the implementation of nationwide nonpharmaceutical interventions (NPIs), including physical distancing, mask use, and school closures, in Japan [17, 18]. These measures are likely to have played a major role in altering the epidemiology of moderate-to-severe RSV infections in Okinawa, particularly with respect to the timing of peak incidence and the age distribution of patients. However, it should be noted that other factors, such as climate variation, differences in circulating viral genotypes, and additional unknown influences, may also have contributed to these changes.

Our study identified several important factors associated with severe RSV infection among hospitalized children in Okinawa. Consistent with prior reports, younger age was a strong predictor of severe RSV infection. This is likely due to the combination of an immature immune system and anatomically narrower airways in infants, which have been demonstrated to contribute significantly to disease severity [19, 20]. The presence of siblings was also an independent risk factor, likely indicating increased exposure to RSV at home through older children [19, 20]. Nursery school attendance was less common among severe cases in the univariate analysis, but this association did not remain after adjustment in the multivariate model (Table 1 and Table 2). This indicates that the effect of nursery school exposure may be influenced by other factors such as age or sibling exposure. Among the 1,541 hospitalized children, 148 (9.6%) were preterm: 46 at ≤ 28 weeks, 76 at 29–35 weeks, and 26 at 36 weeks. Severe cases occurred in 2 (4.3%), 3 (3.9%), and 5 (11.4%) children in these groups, respectively. Although previous studies have shown that preterm infants are at higher risk of severe RSV disease [20], preterm birth was not identified as an independent predictor in our study. This may be partly explained by the limited number of extremely preterm infants and the potential protective effects of prophylaxis, although information on palivizumab or nirsevimab administration was not available in this study. In Japan, infants born at ≤ 28 weeks are generally considered eligible for palivizumab up to 12 months of age, and those born at 29–35 weeks up to 6 months. Moreover, under international recommendations, the majority of preterm infants would have been eligible for nirsevimab prophylaxis, although in Japan its use remains restricted to HRG infants. Underlying diseases were found to significantly increase the risk of severe RSV infection in our study. Although the overall prevalence of underlying diseases did not differ significantly in univariate analysis, the presence of multiple comorbidities was more common among severe cases (Table 1). Multivariate analysis confirmed that both overall and multiple underlying diseases independently increased the risk of severe disease (Table 2). This emphasizes the importance of careful clinical monitoring and prophylactic strategies for children with comorbidities, particularly those with multiple conditions.

The epidemiological pattern of RSV infection in Okinawa differs substantially from that observed in mainland Japan [21–23]. According to the nationwide sentinel surveillance, RSV epidemics in mainland Japan exhibited an annual peak from September to November during the 2012–2015 seasons. In contrast, Okinawa demonstrated broader or bimodal peaks, generally occurring between June and August, approximately six months earlier than in mainland Japan [21]. Between 2016 and 2019, the peak RSV outbreak season in mainland Japan moved from November to around September of the same year [21, 24]. Meanwhile, the timing of the peak in Okinawa remained largely unchanged; however, the peak magnitude gradually increased, resulting in an overlap of the epidemic peaks between Okinawa and mainland Japan [21].

In this study, we analyzed the monthly distribution of moderate-to-severe RSV cases based on medical records from four hospitals between April 2017 and March 2021. From 2017 to 2019, the total cases from the four hospitals showed a clear seasonal peak during the summer months (June–August). In contrast, in 2020, coinciding with the onset of the COVID-19 pandemic, these prominent peaks disappeared (Fig. 2). The temporal trends of moderate-to-severe cases closely matched the overall RSV activity reported for Okinawa, regardless of disease severity [21]. This agreement suggests that moderate-to-severe cases represent a relatively stable portion of the total RSV burden; therefore, monitoring severe cases may serve as a reliable proxy for broader epidemiological patterns and help guide targeted prevention strategies.

Taiwan is located approximately 650 km from the mainland of Okinawa and even closer to the Yaeyama Islands. These regions share similar latitudes, a subtropical to tropical oceanic climate, and comparable patterns in infectious disease epidemiology [25]. In Taiwan, RSV showed no clear seasonality between 1997 and 1999 [26], but from 2015 to 2020, a broad summer peak, especially from July to September, was observed, followed by a winter decline [27]. This pattern closely resembles that of Okinawa, where RSV also peaks in summer [21]. Studies in Thailand from 2015 to 2019 revealed a seasonal trend in RSV-associated ALRTIs, with the highest incidence from August to October during the rainy season [28]. This peak coincides with the peak in hospitalized cases observed in the present study in Okinawa (Fig. 2). Since the seasonal pattern of RSV in Okinawa more closely resembles that of Southeast Asia than mainland Japan, establishing cross-border information-sharing networks should be a priority in regional public health policy to improve the effectiveness of RSV control strategies in Okinawa.

Most hospitalizations for moderate-to-severe RSV infection in this study occurred in infants under 12 months, with a clear peak in the 0–2 month age group—consistent with findings from Europe and other regions [2]—and a secondary peak at 9–11 months (Fig. 3), possibly due to decreasing maternal antibodies and exposure to older siblings. Although hospitalizations decreased after 12 months, a considerable number of cases still required admission up to 24 months (Fig. 3), highlighting the need for continued attention to RSV infection beyond infancy. The age distribution of hospitalized moderate-to-severe RSV cases was consistent across all four study sites, including the remote Miyako and Yaeyama islands, indicating minimal regional variation in severe disease burden within Okinawa Prefecture.

In many countries, the widespread implementation of NPIs during the COVID-19 pandemic has been reported to have markedly suppressed the circulation of respiratory viruses, including RSV [29, 30]. Despite focusing only on hospitalized moderate-to-severe RSV cases, the study found a similar trend: the average annual number of cases declined from 456 pre-pandemic to 172 during the pandemic, about 40% of the previous level (Fig. 4). During the pandemic, the proportion of cases in infants under 6 months increased significantly, while that in the 6–12 months group decreased (Fig. 4). One possible explanation for the observed reduction and age shift during the pandemic is the widespread implementation of NPIs, such as masking, social distancing, and school closures, which significantly suppressed RSV circulation worldwide [29, 31, 32]. At the same time, the continued presence of cases in very young infants indicates that household transmission played a comparatively larger role during this period, leading to the shift in age distribution. Therefore, NPIs may have reduced the overall burden of RSV while indirectly increasing the relative share of infections among younger infants. However, epidemiological changes are rarely attributable to a single factor. Other mechanisms may also have contributed, including temporary reductions in population immunity caused by decreased viral circulation [33], shifts in circulating viral genotypes [22, 34], climatic variations, and region-specific behavioral factors [31]. Although these findings have been reported for RSV infections overall, it is reasonable to assume that the same mechanisms also influenced the subset of children who developed moderate-to-severe disease requiring hospitalization, as observed in our cohort. Taken together, these reports suggest that the unusual RSV epidemic patterns observed in Okinawa during the COVID-19 era were likely influenced by multiple interacting factors, with NPIs playing a key role.

Regarding respiratory support, nearly two-thirds of moderate cases required standard oxygen therapy, while the majority of severe cases required high-flow oxygen or mechanical ventilation. Since ICU admission criteria included the need for advanced respiratory support, these findings reflect the definitions applied for severity classification rather than representing independent associations (Table 3). Overall, 99.8% of patients recovered without any complications, and there were no fatalities (Table 3). Only three patients—all from the severe group—experienced complications: two required prolonged mechanical ventilation, and one developed AESD with developmental delays. The occurrence of these severe outcomes in patients without underlying diseases highlights the potential severity of RSV infection.

Palivizumab has long been the standard method for preventing severe RSV infection; however, it requires monthly injections and is limited to HRG infants [11, 12]. In recent years, nirsevimab has been approved in several countries [13, 14, 35]. As a long-acting monoclonal antibody, it provides season-long protection with a single injection administered before the RSV season and is indicated for all infants regardless of risk status; however, in Japan, insurance coverage is currently limited to infants in HRGs [14]. In parallel, maternal RSV vaccination is being established as a preventive strategy, in which antibodies generated in the mother are transferred to the fetus via the placenta [36]. Taken together, nirsevimab and maternal vaccination are expected to become the primary approaches for preventing severe RSV infection in infants.

Our findings underscore the distinctive epidemiology of RSV in Okinawa, where subtropical climate conditions result in patterns that differ from mainland Japan and more closely resemble Southeast Asia. The comprehensive, entire-prefecture dataset strengthens these observations. By focusing specifically on moderate-to-severe cases, we identified risk factors of direct clinical importance. Additionally, the influence of the COVID-19 pandemic on case numbers and age distribution further emphasizes the need for locally tailored prevention strategies, including considerations for prophylaxis policies such as palivizumab and nirsevimab.

This multicenter epidemiological study covering the entire Okinawa prefecture provides a valuable foundation for understanding the regional burden and characteristics of moderate-to-severe RSV infections.

Limitations include the lack of data on preventive measures such as palivizumab, nirsevimab, and maternal RSV vaccination, as well as the absence of subtype or genotype analysis, since diagnosis depended on rapid antigen testing. Additionally, no standardized research protocol was applied across the four hospitals; although each site followed national standard clinical practices, some variability in diagnostic and clinical procedures cannot be entirely ruled out. Nonetheless, detailed information on seasonality, age distribution, household composition, childcare facility use, and underlying diseases is expected to provide important insights for improving preventive strategies and serve as a basis for assessing and enhancing the implementation of RSV prevention efforts.

## Conclusions

This four-year multicenter study provides a comprehensive epidemiological overview of moderate-to-severe RSV infections among children in subtropical Okinawa, Japan. Approximately 60% of hospitalizations occurred in infants under 12 months, with the highest incidence observed in those aged 0–2 months. Younger age, the presence of siblings, and underlying diseases—particularly when multiple comorbidities were present—were significant independent risk factors for severe disease, whereas preterm birth was not identified as an independent predictor. The summer seasonality contrasts with the winter peaks reported in mainland Japan and aligns more with patterns seen in subtropical and tropical regions. A marked decline and temporal shift in RSV activity during the COVID-19 pandemic further demonstrate the impact of public health measures on viral transmission. These findings provide essential baseline data to guide the development of RSV prevention strategies, including prophylaxis and vaccination policies, tailored to regional epidemiological patterns.

## Data Availability

The data analyzed in this study were extracted from patient medical records maintained at each participating institution and were anonymized to prevent personal identification. In accordance with institutional policies and ethical regulations, the dataset cannot be made publicly available.

## Abbreviations

RSV: Respiratory syncytial virus
ALRTIs: Acute lower respiratory tract infections
HRGs: High-risk groups
MSAs: Medical service areas
ONMC: Okinawa Nanbu Medical Center & Children’s Medical Center
ICU: Intensive care unit
IQR: Interquartile range
OR: Odds ratio
CI: Confidence interval
AESD: Acute encephalopathy with biphasic seizures and late reduced diffusion
NPI: Non-pharmaceutical intervention
